# *Dichotomius* (*Luederwaldtinia*) *schiffleri* (Coleoptera: Scarabaeidae) mitochondrial genome and phylogenetic relationships within the superfamily Scarabaeoidea

**DOI:** 10.1080/23802359.2017.1407695

**Published:** 2017-11-27

**Authors:** Igor Costa Amorim, Adriana de Souza Melo, Geyner Alves dos Santos Cruz, Gabriel da Luz Wallau, Rita de Cássia de Moura

**Affiliations:** a Laboratório de Biodiversidade e Genética de Insetos – Instituto de Ciências Biológicas, Universidade de Pernambuco, Recife, PE, Brasil;; b Departamento de Entomologia, Instituto Aggeu Magalhães – Fundação Oswaldo Cruz, Recife, PE, Brasil

**Keywords:** Mitogenome, nucleic acid and protein phylogeny, mtDNA organization

## Abstract

The mitochondrial DNA of *Dichotomius* (*Luederwaldtinia*) *schiffleri* was characterized and its phylogenetic position was reconstructed in Scarabaeoidea. This mitogenome presented 14,802 bp-long, richness in AT of 77.4% and 37 genes, including 13 protein-coding, 22 transfer RNAs, and two ribosomal RNAs. In addition, it was observed intergenic spacers and reading frame overlaps. The phylogenetic trees reconstructed from protein sequences provided best resolution, indicating Scarabaeinae and Aphodiinae as a sister groups, as previously reported in other molecular phylogenies.

*Dichotomius* (*Luederwaldtinia*) *schiffleri* Vaz-de-Mello, Louzada, Gavino 2001 is an Endangered species, endemic to Brazilian coastal ecosystems (Vieira et al. 2011). Here it was characterized the mtDNA of *D.* (*L.*) *schiffleri* and established its position in the superfamily Scarabaeoidea based on phylogenomic methods.

The specimen analyzed was collected in Ipojuca (8°31'26" S 35°1'31" W), Pernambuco, Brazil and deposited in the Laboratório de Biodiversidade e Genética de Insetos (accession number: LBGI_M9032) at the Universidade de Pernambuco, Brazil. The collection of this species was authorized by IBAMA/SISBIO (Licence No. 50438-1). The DNA was extracted according to the protocol of Sambrook and Russel (2001). The mitogenome was sequenced on Solexa-Illumina HiSeq 2000 (San Diego, CA). The genome assembly was performed with the Oligonucleotide Analysis Package, screened through a reference mitogenome and characterized on the MITOS WebServer. For phylogenetic analysis, the complete or partially mitogenome sequence of species of Scarabaeoidea superfamily from GeneBank was used. The Staphyliniformia species were used as outgroup. The phylogenetic trees of nucleic acid and protein sequences were reconstructed by Bayesian inference and maximum likelihood methods.

The mitogenome of *D.* (*L.*) *schiffleri* comprised a coding region of 14,802 bp (accession number: KY100258), with high percentage of A-T (77.4%). The characterization revealed the presence of 37 genes, including 13 protein-coding (cox1-3, cob, nad1-6, nad4L, atp6, and atp8), 22 tRNA, and two rRNA genes (rrnS and rrnL). Regarding the orientation, 23 genes are located on the positive chain and 14 genes on negative chain. Mitogenome size, nucleotide composition, number, orientation, and order of genes are similar to most of the insects (Sheffield et al. 2008, 2009; Cameron 2014). In the mitogenome of *D.* (*L.*) *schiffleri* 23 intergenic spacers and six reading frame overlaps were observed, including an overlap of 36 bp between the *rrnL* and *trnL1* genes, corresponding to more than half of the sequence of this tRNA (67 bp). The presence of intergenic spacers and reading frame overlaps was also observed in other Coleoptera species (Sheffield et al. 2008).

Regarding the phylogenetic analysis the Bayesian and maximum likelihood trees presented similar topology. However, the trees reconstructed from protein sequences showed a better resolution and correctly positioned the species/family (Figure 1 and Supplemental Figure S1), what was previously reported in other analyses in Coleoptera (Du et al. 2016; Timmermans et al. 2016). Therefore, here it was discussed only the protein tree. The phylogeny showed *D.* (*L.*) *schiffleri* on the same clade as *Sarophorus* sp. with the subfamily Aphodiinae as a sister group (Figure 1, subclade A). The position of Aphodiinae as a sister group of Scarabaeinae was observed in other phylogenies based on nuclear and mitochondrial genes (Bocak et al. 2014; Timmermans et al. 2016). In turn, the superfamily Scarabaeoidea formed a monophyletic group (Figure 1, clade I). The characterization of the mitogenome of *D.* (*L.*) *schiffleri* provides useful knowledge for future studies aimed to investigate the genetic diversity on population level and develop strategies for the management and conservation of this species.

**Figure 1.**
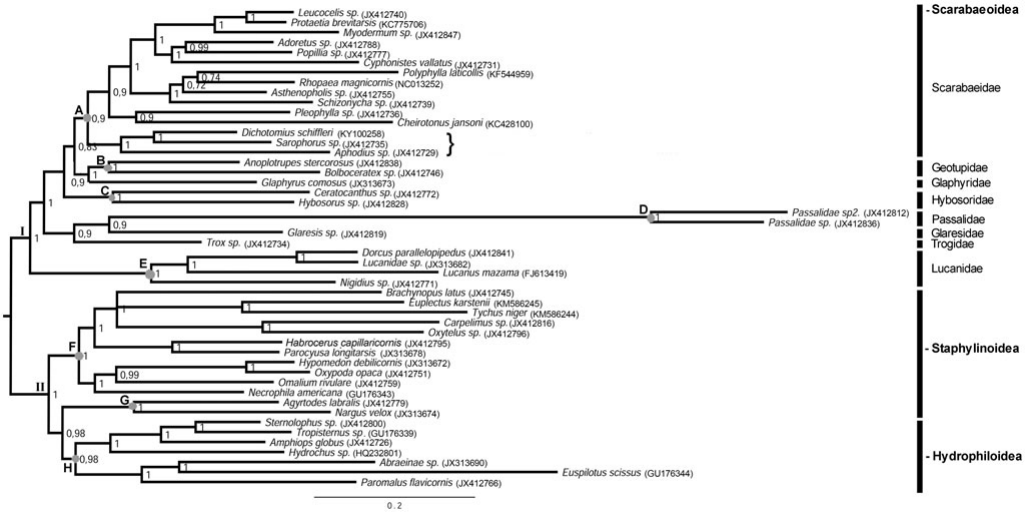
Bayesian inference of species of the superfamily Scarabaeoidea based on 13 protein-coding genes of the mitochondrial genomes. Species of Staphylinoidea and Hydrophiloidea, available in GenBank, were used as outgroup. The multiple bars indicate the different Scarabaeoidea families (clade I) and the superfamilies Staphylinoidea and Hydrophiloidea (clade II). Species highlighted in closed brace belong to the subfamilies Scarabaeinae and Aphodiinae. The highlighted nodes indicate subclades with species from the same family or superfamily. In the nodes (A) Scarabaeidae; (B) Geotupidae (C) Hybosoridae; (D) Passalidae; (E) Lucanidae; (F and G) Staphylinoidea superfamily; (H) Hydrophiloidea superfamily.

## Supplementary Material

Rita_C_ssia_Moura_et_al_supplemental_content.zip
