# Supplementary material for: Dichotomius (Luederwaldtinia) schiffleri (Coleoptera: Scarabaeidae) mitochondrial genome and phylogenetic relationships within the superfamily Scarabaeoidea
Source: Mitochondrial DNA B Resour. 2017 Nov 27;2(2):887–8. doi: 10.1080/23802359.2017.1407695 (PMC7800706; doi:10.1080/23802359.2017.1407695)
Supplement: Rita_C_ssia_Moura_et_al_supplemental_content.zip [file TMDN_A_1407695_SM3523.zip › Rita Cássia Moura et al supplemental content.pdf]

Supplemental data for the manuscript:

***Dichotomius* (*Luederwaldtinia*) *schiffleri* (Coleoptera: Scarabaeidae) mitochondrial genome and phylogenetic relationships within the superfamily Scarabaeoidea**

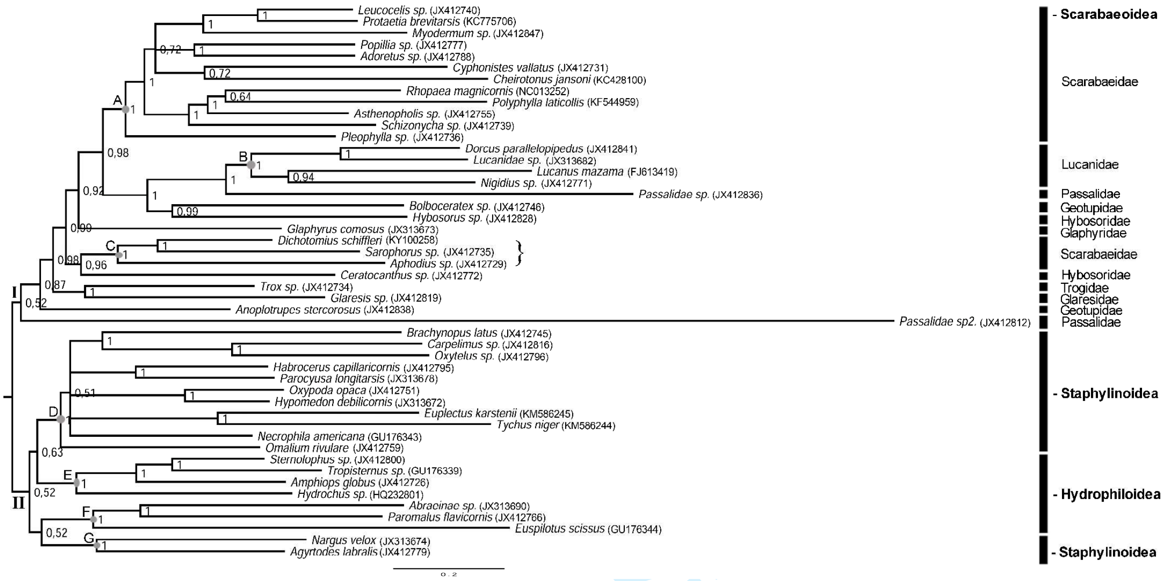

Supplemental Figure S1. Bayesian inference of species of the superfamily Scarabaeoidea based on the nucleotide sequences of the mitogenoma. The multiple bars indicate the different Scarabaeoidea families (I) and the superfamilies Staphylinoidea and Hydrophiloidea (II). Species highlighted in closed brace belong to the subfamilies Scarabaeinae and Aphodiinae. The highlighted nodes indicate subclades with species from the same family or superfamily. In the nodes (A and C) Scarabaeidae; (B) Lucanidae; (D and G) Staphylinoidea superfamily; (E and F) Hydrophiloidea superfamily.

For Peer Review Only
